# Pyroptosis, apoptosis, and autophagy are involved in infection induced by two clinical *Klebsiella pneumoniae* isolates with different virulence

**DOI:** 10.3389/fcimb.2023.1165609

**Published:** 2023-05-08

**Authors:** Xueting Wang, Chunxia Bi, Xiaoni Xin, Mengmeng Zhang, Hengxia Fu, Lei Lan, Mengyuan Wang, Zhiyong Yan

**Affiliations:** ^1^ Institute of Medical Faculty, Qingdao University, Qingdao, China; ^2^ Department of Clinical Laboratory, Qingdao Municipal Hospital, Qingdao, China; ^3^ Department of Clinical Laboratory, Shandong Provincial Second People’s Hospital, Jinan, China; ^4^ Department of Clinical Laboratory, Linyi Central Hospital, Linyi, China; ^5^ Department of Blood Transfusion, Qingdao Women and Children’s Hospital, Qingdao, China; ^6^ Department of Clinical Laboratory, Jinan Children’s Hospital, Jinan, China; ^7^ College of Basic Medicine, Medical Faculty of Qingdao University, Qingdao, China

**Keywords:** inflammation, pyroptosis, apoptosis, autophagy, macrophage, *Klebsiella pneumoniae*

## Abstract

*Klebsiella pneumoniae* can cause widespread infections and is an important factor of hospital- and community-acquired pneumonia. The emergence of hypervirulent *K. pneumoniae* poses a serious clinical therapeutic challenge and is associated with a high mortality. The goal of this work was to investigate the influence of *K. pneumoniae* infection on host cells, particularly pyroptosis, apoptosis, and autophagy in the context of host–pathogen interactions to better understand the pathogenic mechanism of *K. pneumoniae*. Two clinical *K. pneumoniae* isolates, one classical *K. pneumoniae* isolate and one hypervirulent *K. pneumoniae* isolate, were used to infect RAW264.7 cells to establish an *in vitro* infection model. We first examined the phagocytosis of macrophages infected with *K. pneumoniae*. Lactate dehydrogenase (LDH) release test, and calcein-AM/PI double staining was conducted to determine the viability of macrophages. The inflammatory response was evaluated by measuring the pro-inflammatory cytokines and reactive oxygen species (ROS) production. The occurrence of pyroptosis, apoptosis, and autophagy was assessed by detecting the mRNA and protein levels of the corresponding biochemical markers. In addition, mouse pneumonia models were constructed by intratracheal instillation of *K. pneumoniae* for *in vivo* validation experiments. As for results, hypervirulent *K. pneumoniae* was much more resistant to macrophage-mediated phagocytosis but caused more severe cellular damage and lung tissues damage compared with classical *K. pneumoniae*. Moreover, we found increased expression of NLRP3, ASC, caspase-1, and GSDMD associated with pyroptosis in macrophages and lung tissues, and the levels were much higher following hypervirulent *K. pneumoniae* challenge. Both strains induced apoptosis *in vitro* and *in vivo*; the higher apoptosis proportion was observed in infection caused by hypervirulent *K. pneumoniae*. Furthermore, classical *K. pneumoniae* strongly triggered autophagy, while hypervirulent *K. pneumoniae* weakly activated this process. These findings provide novel insights into the pathogenesis of *K. pneumoniae* and may form the foundation for the future design of treatments for *K. pneumoniae* infection.

## Introduction

*Klebsiella pneumoniae* is a gram-negative opportunistic pathogen and frequently causes a number of illnesses in hospitalized or otherwise immunocompromised patients, including pneumonia, urinary tract infection, and bacteremia, posing a serious threat to public health worldwide (Martin and Bachman, 2018). In China, *K. pneumoniae* accounted for 11.9% of bacterial isolates from ventilator-associated pneumonia and ICU-acquired pneumonia (Zhang et al., 2014). According to incomplete statistics, co-infection with *K. pneumoniae* was the second most prevalent among coronavirus disease 2019 (COVID-19) patients, following *Streptococcus pneumoniae* (Zhu et al., 2020b). Additionally, the rate of multidrug resistance of *K. pneumoniae* increased gradually, causing a difficulty in clinical treatment (Wang et al., 2020b). Over the past several decades, a virulent subtype known as hypervirulent *K. pneumoniae* (hvKp) responsible for both community- and hospital-acquired infection has emerged worldwide. In contrast to classical *K. pneumoniae* (cKp), this strain, especially K1 and K2 serotypes, can cause more severe and metastatic infections such as pyogenic liver abscesses, endophthalmitis, and meningitis even in immunocompetent hosts of any age (Russo and Marr, 2019). The presence of genotypic markers iucA, peg-344, and rmpA can be used, albeit imperfectly, to most accurately distinguish the cKp and hvKp strains as previously reported (Nicolò et al., 2022). There was a high prevalence of hvKp among *K. pneumoniae* isolates ranging from 31% to 37.8% in China (Zhang et al., 2016). Although the incidence of pneumonia induced by hvKp was only approximately 3%–8% of all pneumonia, the 5-year mortality rate achieved 40%–60% (Lee et al., 2017b; Russo and Marr, 2019). It was a serious threat deserving clinical attention. The development of novel approaches to treat *K. pneumoniae* infection is urgent, and immunotherapy that targets the anti-immune strategies of this pathogen could be a promising strategy (Wei et al., 2022). Understanding the mechanism of host–pathogen interactions is a prerequisite for the development of this strategy. However, whether or how *K. pneumoniae* affects host innate immune system has not been fully elucidated.

Macrophages are one of the most important innate immune cells, performing crucial roles in the host defense against microbial infections *via* the secretion of a lot of inflammatory mediators such as NO, ROS, and cytokines [tumor necrosis factor alpha (TNF-α), interleukin (IL)-6, IL-12, and IL-1β] (Tosi, 2005; Arango Duque and Descoteaux, 2014; Galli and Saleh, 2020). The initial phagocytes to encounter *K. pneumoniae* on the mucosal surface of lung tissues are macrophages (Aberdein et al., 2013). Although macrophage plays a crucial role in clearing bacterial infection, the macrophage responses after *K. pneumoniae* infection are poorly characterized. It has been reported that autophagy, inflammation, and cell death are critical host defense mechanisms against invading bacteria (Bengoechea and Sa Pessoa, 2019; Wei et al., 2022).

Inflammatory caspases (caspase-1, caspase-11, caspase-4, and caspase-5) are essential for the innate immune defense. Numerous inflammasomes, including NLRP3, NLRP1, NLRC4, AIM2, and Pyrin, can activate caspase-1 (Lamkanfi and Dixit, 2014). When caspase-1 is activated, it will cause the release of IL−1β and IL−18 and a specific type of inflammatory necrosis known as pyroptosis, characterized by the formation of membrane pores, cell swelling, and plasma membrane rupture, ultimately resulting in massive leakage of cell contents and a strong inflammatory reaction (Cookson and Brennan, 2001; Broz and Dixit, 2016). Accumulating studies indicate that pyroptosis is associated with various inflammatory diseases, such as infectious diseases, nervous diseases, and atherosclerotic diseases (Lamkanfi, 2011; Strowig et al., 2012; Latz et al., 2013). Furthermore, as a cell defense and survival mechanism, autophagy can deliver cellular components or invading microorganisms by double membrane vesicles called autophagosomes to lysosomes for degradation, which is critical for maintaining cellular homeostasis. The autophagy process contains three steps: formation of double membrane autophagosomes, autophagosome fusion with lysosome, and cargo degradation (Yang and Klionsky, 2010). Previous research showed that autophagy mediated bacterial clearance during *Pseudomonas aeruginosa* infection (Li et al., 2015b; Zhu et al., 2020a). Notably, recent studies have shown that autophagy could downregulate the inflammatory response in macrophages by clearing the excessive ROS (Saitoh and Akira, 2016). It is now widely acknowledged that a number of bacteria disrupt the autophagy process (Deretic et al., 2013). Impaired autophagy was extensively involved in various pathological processes of many diseases (Levine and Kroemer, 2008).

In this study, we selected clinical cKp and hvKp strains to establish macrophage infection *in vitro* and mouse pneumonia *in vivo* models, respectively, in order to examine the impacts of these two strains infection on host cells. We mainly focused on the host–pathogen interactions in aspects of pyroptosis, apoptosis, and autophagy. Our results will provide a theoretical basis to design novel effective therapeutic strategies for *K. pneumoniae* infection.

## Materials and methods

### Cell culture and bacterial strains

RAW264.7 cells (leukemia cells in mouse macrophage) were purchased from American Type Culture Collection (TIB-71, VA, USA) and grown in Dulbecco’s modified Eagle’s medium with high glucose (VivaCell, Shanghai, China), which contains 10% fetal bovine serum (TransGen, Beijing, China). The medium was antibiotic-free during cell culture. Cells were cultured in a sterile and humidified incubator with 5% CO_2_ at 37°C.

One classical *K. pneumoniae* isolate and one hypervirulent *K. pneumoniae* isolate were obtained from the Laboratory Medicine at the Affiliated Hospital of Qingdao University (Qingdao, China). Bacteria were inoculated in lysogeny broth (LB) medium and cultivated at 37°C and 220 rpm with shaking overnight. After centrifuging for 5 min at 12,000 rpm, the bacteria pellets were collected and resuspended in sterile phosphate-buffered saline (PBS) for subsequent infection experiments. PBS was served as blank control. A summary of the genetic information of the studied strains is listed in **Supplementary Table S1** and **Figures S1**, **S2**.

### Phagocytosis analysis

Phagocytosis of fluorescein isothiocyanate (FITC)-labeled *K. pneumoniae* by macrophages was assessed as previously described (Li et al., 2008), with slight modifications. First, the overnight cultured bacterial suspension was soaked in a 60°C water bath for 15 min to kill the bacteria. Following that, bacterial particles were washed twice with PBS, with the concentration adjusted to 1×10^8^ colony forming units (CFU)/ml in PBS. Then, each 1-ml bacteria suspension was mixed with 20 μl of 5 mg/ml FITC (Solarbio, Beijing, China) and incubated for 1 h under dark. After three washes, FITC-labeled bacteria were resuspended at a concentration of 1×10^9^ CFU/ml in PBS. For phagocytosis, a mixture of 1-ml macrophage suspension (containing 1×10^6^ cells) and 50 μl FITC-labeled cKp or hvKp suspension (representing 5×10^7^ cells) was incubated at room temperature for 1 h away from light and shaken every 10 min. The non-ingested bacteria were separated from macrophages by centrifugation at 1,000 rpm for 10 min. Only the intracellular bacteria fluorescence can be detected by quenching the extracellular bacteria fluorescence with 0.4% trypan blue. A total of 30,000 cells were processed by flow cytometry (Beckman Coulter, CA, USA).

### Colony forming units counting

RAW264.7 cells were infected with cKp or hvKp at a multiplicity of infection (MOI) of 50:1 for 1 h at 37°C, 5% CO_2_ in an antibiotic-free medium. After that, cells were washed three times with PBS and incubated for additional 1 h in a medium containing 50 µg/ml gentamicin. Then, cells were washed and subsequently lysed with 0.2% Triton X-100 (in PBS). Upon homogenization, 10-fold serial dilutions of the lysate were plated on LB agar plates incubating overnight at 37°C to determine the number of CFUs.

### Cell viability analysis

RAW264.7 cells were seeded in 24-well plates with 1×10^5^ cells per well and continuously incubated overnight to promote cell adherence. Then, cells were infected with cKp and hvKp strains for additional 24 h, respectively, with an MOI of 10:1. After infection, cells were washed three times with PBS, and the viability of cells was detected by a Calcein-AM/PI double staining kit (Meilunbio, Shanghai, China) in accordance with manufacture protocols. Briefly, 300 μl of staining assay buffer with 2 µM calcein−AM and 8 µM PI were added to each well and incubated at 37°C for 20 min protected from light, and the results were examined by an inverted fluorescence microscope (Olympus, Tokyo, Japan). The PI-positive cells are red and represented dead cells.

### LDH release analysis

The activity of lactate dehydrogenase (LDH) was detected by a LDH release assay kit (Yeasen, Shanghai, China) according to the instructions of the test kit. Macrophages were seeded in 96-well plates (1×10^4^ cells per well) and cultured overnight. Next day, cells were washed two times with PBS and added fresh medium containing 1% fetal bovine serum. Then, the PBS, cKp, and hvKp treatments were applied for an additional 24 h, respectively. The sample maximum enzyme activity control wells did not undergo any treatment, and cell lysis buffer (10% of the medium volume) was added 1 h before testing and continued incubation. Next, 120 μl of cell supernatant was harvested and added to a new 96-well plate. A total of 60 μl of LDH detection solution was added to each well and incubated at room temperature for 30 min. The absorbance value at 492 nm was detected by a microplate reader (BioTek, VT, USA), which is positively correlated with LDH release. The percentage of LDH release (%) was calculated by the formula: (absorbance of PBS or cKp or hvKp treated sample/absorbance of cell lysis buffer treated sample) × 100%

### ELISA analysis

Macrophages were infected with cKp and hvKp strains at an MOI of 10:1 for 24 h, respectively. Cell culture medium was collected and centrifuged for 15 min at 4°C, 3,000 rpm. Then, the supernatant was harvested for further analysis. Quantitative analysis of IL-1β, IL-6, and TNF-α in supernatant was performed by ELISA kits (Invitrogen, CA, USA), in line with manufacturer’s recommendations. The optical density (OD) values of every well were examined at 450 nm using the microplate reader (BioTek, VT, USA). Meanwhile, the standard curve was fitted according to the standard concentration and absorbance, and then, the sample concentration was calculated from the standard curve equation.

### ROS detection

Macrophages were seeded in six-well plates with 5×10^5^ cells per well. Cells were infected with either cKp or hvKp for 24 h (MOI=10:1) at 37°C, 5% CO_2_ in DMEM high glucose medium containing 10% fetal bovine serum (FBS), and PBS treatment was served as control. Following this, DCFH-DA fluorescent probe was diluted at 1:2,000 with serum-free medium and co-incubated with cells at 37°C for 25 min under dark. Then, cells were washed three times with PBS to remove extracellular DCFH-DA. Finally, the cells were immediately viewed by an inverted fluorescent microscope (Olympus, Tokyo, Japan) or collected and detected by flow cytometry (Beckman Coulter, CA, USA). FlowJo software (version 10, USA) was used to analyze the date.

### Quantitative real-time PCR

RNAiso Plus kit (Takara, Kyoto, Japan) was used to extract total RNA from RAW264.7 cells and lung tissues. After measuring the concentration and purity of RNA, complementary DNA was generated by cDNA synthesis kit (Yeasen, Shanghai, China), and SYBR Green qPCR master mix (Vazyme, Nanjing, China) was used to quantify the RNA levels. Amplification reactions were carried out on a CFX96 real-time quantitative PCR instrument (Bio-Rad, CA, USA), and each reaction was performed in triplicate. The relative expression of genes was calculated using the 2^−ΔΔCT^ method, and β-actin gene served as control for standardization. The primer sequences are described in **Table 1**.

**Table 1:** Primers used in this study.

Gene	Primer sequence (5′ to 3′)
IL-1β	Forward: GCCACCTTTTGACAGTGATGAG Reverse: CCTGAAGCTCTTGTTGATGTGC
TNF-α	Forward: GTCTACTGAACTTCGGGGTGA Reverse: CTCCTCCACTTGGTGGTTTG
IL-6	Forward: CTTCTTGGGACTGATGCTGGTGAC Reverse: AGTGGTATCCTCTGTGAAGTCTCCTC
NLRP3	Forward: GGACAGCCTTGAAGAAGAGTGGATG Reverse: CTGCGTGTAGCGACTGTTGAGG
GSDMD	Forward: CGATGGGAACATTCAGGGCAGAG Reverse: ACACATTCATGGAGGCACTGGAAC
Caspase-1	Forward: GCCGTGGAGAGAAACAAGGAGTG Reverse: TCAATGAAAAGTGAGCCCCTGACAG
Bcl-2	Forward: CCAGCCTGAGAGCAACCCAATG Reverse: ACGACGGTAGCGACGAGAGAAG
Bax	Forward: TGCAGAGGATGATTGCTGAC Reverse: GATCAGCTCGGGCACTTTAG
Caspase-3	Forward: TCTGACTGGAAAGCCGAAACTCTTC Reverse: GTCCCACTGTCTGTCTCAATGCC
LC3	Forward: CAAGCCTTCTTCCTCCTGGTGAATG Reverse: CCATTGCTGTCCCGAATGTCTCC
Beclin-1	Forward: GACGAACTCAAGAGTGTGGAGAACC Reverse: AGATGTGGAAGGTGGCATTGAAGAC
β-actin	Forward: GCAGCTCAGTAACAGTCCGC Reverse: AGTGTGACGTTGACATCCGT

### Western blotting

Total proteins were extracted from macrophages with RIPA lysis buffer (Solarbio, Beijing, China) containing 1% protease inhibitor cocktail (Meilunbio, Shanghai, China) to prevent protein degradation. After the protein concentration was measured by BCA kit (Meilunbio, Shanghai, China), 5× loading buffer was added and boiled for 10 min to fully denature the protein. Equal masses of proteins were separated by 12.5% sodium dodecyl sulfate–polyacrylamide gel electrophoresis (SDS-PAGE) gels and transferred to polyvinylidene difluoride (PVDF) membranes (Millipore, MA, USA). Membranes were incubated with primary antibodies overnight at 4°C after blocking with 5% non-fat powdered milk at room temperature for 2 h. The next day, membranes were washed with Tris-buffered saline with Tween 20 (TBST) three times before incubation with horseradish peroxidase (HRP)-conjugated secondary antibodies. Then, the membrane bands were detected using enhanced chemiluminescence reagent (Epizyme, Shanghai, China) and analyzed by ImageJ software (version 8, National Institutes of Health). The primary antibodies were as follows: anti-LC3A/B (1:1,500, Cell Signaling Technology, USA), anti-caspase-1 (1:1,500, Abclonal, China), anti-GSDMD (1:1,500, Abcam, UK), anti-NLRP3 (1:1500, Abclonal, China), and anti-β-actin (1:200,000, Abclonal, China).

### Immunofluorescence

Cells (1×10^5^) were cultured on coverslips in 24-well plates and continuously treated with different stimuli for 24 h. Next, cells were sequentially fixed and permeabilized with 4% paraformaldehyde and 0.5% Triton X-100. Following a 30-min blocking step with normal goat serum, cells were treated overnight at 4°C with anti-LC3A/B antibodies. The next day, cells were incubated with Alexa Fluor 647-labeled secondary antibodies for 1 h at room temperature away from light after three TBST washes. Finally, the coverslips were sealed with an anti-fluorescence quenching sealer containing 4′,6-diamidino-2-phenylindole (DAPI). Fluorescence images were obtained on a fluorescence microscope (Zeiss, Oberkochen, Germany).

### Apoptosis analysis

Cells were collected 24 h after the indicated treatments, washed twice with cooled PBS, resuspended in 1× binding buffer, and adjusted for cell concentration to 1×10^6^ cells/ml. Cell suspensions (100 μl) were mixed with 5 μl Annexin V-FITC and 5 μl PI and incubated at room temperature for 15 min, then added with 400 μl PBS and immediately detected using flow cytometry (Beckman Coulter, CA, USA). Gating strategy was applied to analyze the apoptosis of macrophages.

### Animal experiments

In this study, wild-type male C57BL/6 mice at 8–10 weeks old were purchased from Sibeifu biotechnology Co., Ltd. (Beijing, China). Mice were kept in a specific pathogen-free environment with a 12-h light/dark cycle at 23°C–25°C and humidity of 35%–75% and received enriched water and *ad libitum* feeding. All mice were adapted in this environment for 1 week. The mice were randomly assigned into the PBS control group, the cKp group and the hvKp group with five mice in each group. For *in vivo* experiments, mice were anesthetized by an intraperitoneal injection of 1% pentobarbital sodium (50 mg/kg) and then infected with an intratracheal administration of cKp or hvKp suspension (1×10^5^ CFU suspended in 50 μl sterile PBS), respectively. The dosage design of bacteria was based on previous studies. Control mice were intratracheally administered with an equal volume of sterile PBS. Briefly, mice were fixed on a triangular frame in an upright position after anesthesia. The tongue was gently pulled out from the mouth and the 22-G catheter was inserted into the trachea along the tongue’s root. It was made sure that the insertion was not too deep because if it was, fluid will unilaterally be injected into either the right or left major bronchus. Then, mice were held in an upright position and rotated so that the bacterial suspension distributed evenly throughout each lung. At 24 h after treatment, mice were sacrificed, and the chest cavity was opened to expose the lungs. The right ventricle was punctured with syringes containing 10 ml pre-cooled PBS, and the pulmonary circulation was flushed twice with PBS. Next, fresh lung tissues were obtained, the left lung tissues were stored at −80°C for RNA extraction and further gene expression analysis, and the right lung tissues were immediately fixed with 4% paraformaldehyde (PFA) for 48 h for histological evaluation. All experimental procedures were conducted in strict accordance with the animal welfare ethics requirements of Qingdao University and approved by the Laboratory Animal Welfare Ethics Committee of Qingdao University (No. 20220825C575120220921133).

### Survival analysis

Mice (12 mice in each group) were treated as described above and closely monitored everyday for the signs of hunched posture, ruffled fur, difficulty of breathing, reluctant to move, photophobia, and dehydration. Mice that developed these symptoms were humanely euthanized and counted as dead. Survival rates were measured continuously for 7 days in each group and recorded every 24 h.

### Hematoxylin and eosin staining

The fixed lung tissues were embedded in paraffin and cut into 5-µm sections. Then, sections were stained with hematoxylin and eosin for the pathological changes assessment, followed by an observation with a light microscope (Nikon, Tokyo, Japan).

### Immunohistochemistry staining

Immunohistochemistry procedures were performed as what a former report depicted with minor modifications (Zhang et al., 2022). Briefly, after deparaffinization and rehydration, the lung sections were immersed in 1× sodium citrate buffer at 98°C for 15 min to repair antigen, then treated with 3% hydrogen peroxide for 10 min at room temperature to inactivate endogenous peroxidase. Subsequently, sections were blocked with goat serum for 1 h and then incubated with primary antibodies against F4/80, NLRP3, ASC, LC3A/B, and cleaved caspase-3 overnight at 4°C, followed by the secondary antibodies at room temperature for 30 min. Finally, all sections were stained with DAB reagent kit (Solarbio, Beijing, China) and then counterstained with hematoxylin. All sections were observed using an optical microscope (Nikon, Tokyo, Japan).

### Statistical analysis

All statistical analyses were performed using GraphPad Prism 9.0 (GraphPad Software, USA). Data were presented as mean ± standard deviation (SD), and comparisons between groups were performed by one-way analysis of variance (ANOVA) followed by Tukey’s multiple comparisons test or Welch’s ANOVA followed by Dunnett’s T3 multiple comparisons test. *p* < 0.05 was considered statistically significant.

## Results

### Phagocytosis of *K. pneumoniae* by macrophages

**Figure 1:**
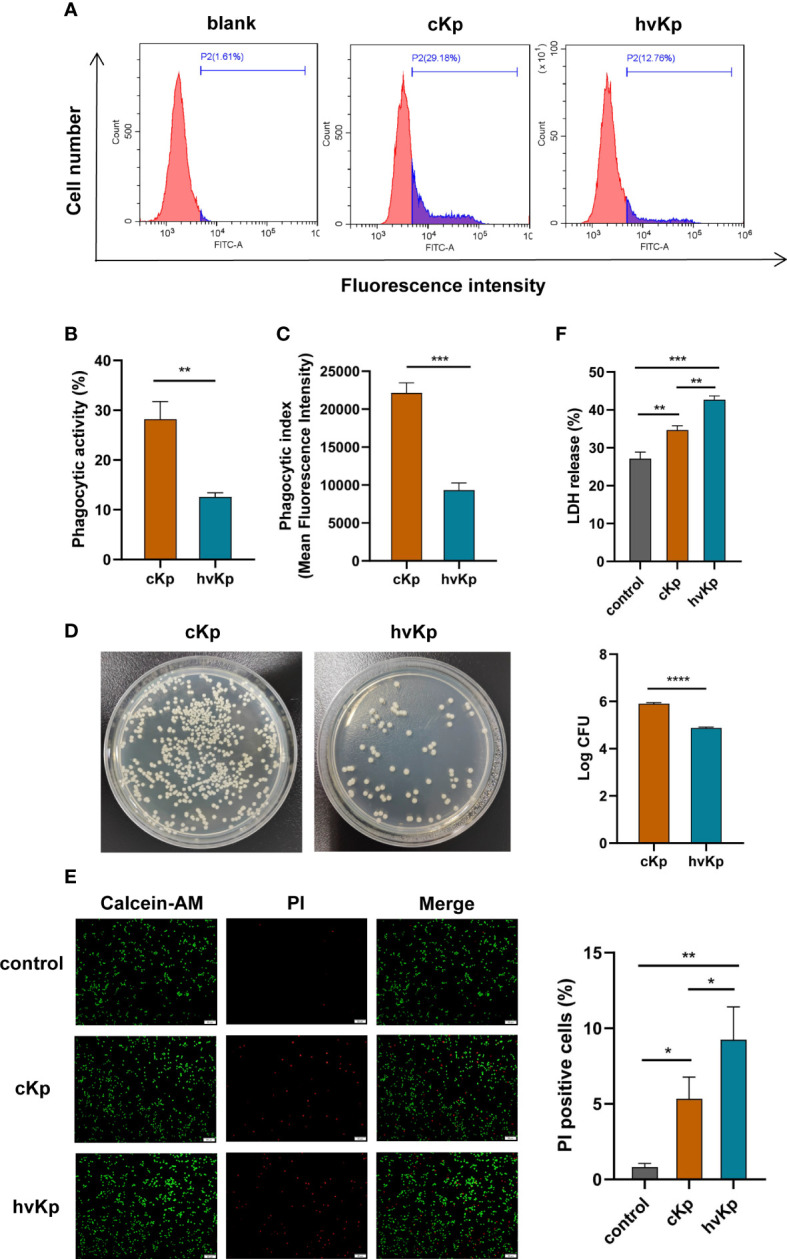
Effects of cKp and hvKp infection on phagocytic capability and cell viability of macrophages. **(A)** The histograms of flow cytometric analysis of phagocytosis in RAW264.7 cells treated by cKp and hvKp. **(B)** Phagocytic ability (PA) values of RAW264.7 cells. **(C)** Phagocytic index (PI) values of RAW264.7 cells. **(D)** Bacterial load of macrophages was detected by colony forming units counting. **(E)** Calcein−AM/PI double staining assay was performed for living cells (green fluorescence) and dead cells (red fluorescence) (scale bar, 50 μm). **(F)** The levels of LDH release in RAW264.7 cells. All experiments were independently repeated three times. Data are presented as mean ± SD. *p < 0.05, **p < 0.01, ***p < 0.001, ****p < 0.0001. cKp, classical *Klebsiella pneumoniae*; hvKp, hypervirulent *Klebsiella pneumoniae*.

To assess the phagocytic capability of macrophages, FITC-labeled cKp and hvKp strains were co-incubated with macrophages, respectively. As shown in **Figures 1A, B**, the phagocytic activity (PA) of macrophages in cKp group was much higher, with an average of 28.2% (range, 24.2%–31.1%), while the average was only 12.6% (range, 11.6%–13.3%) in the hvKp group. Consistent with PA results, the phagocytic index (PI) of macrophages engulfing cKp was also much higher compared with that of the macrophages engulfing hvKp (**Figure 1C**). Additionally, the bacterial load (CFUs) in the hvKp group was much lower than that in the cKp group (**Figure 1D**). To sum up, these data indicated that the hvKp strain was significantly more resistant to macrophage-mediated phagocytosis than cKp strain.

### *K. pneumoniae* infection decreases cell viability and increases LDH release from macrophages

In order to explore the cytotoxicity of *K. pneumoniae* on macrophages, we tested the cell viability and LDH release from RAW264.7 cells following *K. pneumoniae* infection. Results from the calcein−AM/PI double staining assay displayed that the viability of macrophages infected with cKp and hvKp was greatly decreased compared with the control group (**Figure 1E**). In comparison with the cKp strain, hvKp strain markedly lowered the cell viability. In addition, LDH release was taken as an indicator of lytic cell death. As shown in **Figure 1F**, compared to the control group, LDH activities in the cell culture supernatant of either the cKp group or hvKp group were both considerably higher. Moreover, the hvKp strain also caused greater release of LDH than the cKp strain. These findings demonstrated that *K. pneumoniae* infection induced cytotoxicity in macrophages, and the hvKp strain can cause more serious cell damage than the cKp strain.

### *K. pneumoniae* infection increases the production of pro-inflammatory cytokines and ROS in macrophages

**Figure 2:**
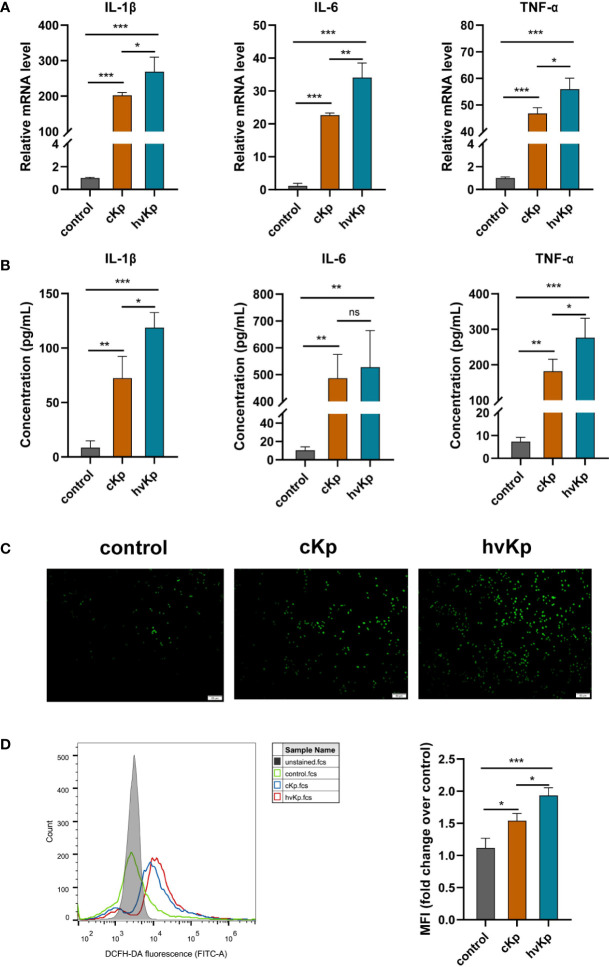
Effects of cKp and hvKp infection on the production of pro-inflammatory cytokines and ROS in macrophages. **(A)** Relative mRNA expression of IL-1β, IL-6, and TNF-α in RAW264.7 cells by qRT-PCR. **(B)** The protein concentrations of IL-1β, IL-6, and TNF-α in cell supernatant by ELISA. **(C)** Fluorescence images of ROS accumulation in RAW264.7 cells (scale bar, 50 μm). **(D)** The quantitative analysis of ROS production in RAW264.7 cells by flow cytometry. All experiments were independently repeated three times. Data are presented as mean ± SD. ns, no significance, *p < 0.05, **p < 0.01, ***p < 0.001. cKp, classical *Klebsiella pneumoniae*; hvKp, hypervirulent *Klebsiella pneumoniae*.

The levels of pro-inflammatory cytokines IL-1β, IL-6, and TNF-α were detected using qRT-PCR and ELISA methods, respectively. Results of qRT-PCR demonstrated that compared to the control group, the mRNA levels of these pro-inflammatory cytokines were markedly upregulated with *K. pneumoniae* stimulation. Moreover, there was a much higher mRNA expression of these pro-inflammatory cytokines in the hvKp group than that in the cKp group (**Figure 2A**). Additionally, we further measured the concentrations of these pro-inflammatory cytokines in cell culture medium. ELISA analysis showed that both cKp and hvKp strains dramatically promoted the release of IL-1β, IL-6, and TNF-α in macrophages (**Figure 2B**). Similarly, hvKp strain significantly increased the IL-1β and TNF-α release in macrophages compared with cKp strain. However, there was no statistically significant difference between cKp and hvKp groups for the IL-6 concentration.

During infection, macrophages will release excessive ROS, leading to cellular damage and even cell death. In the current study, we wondered whether cKp and hvKp infection modulate the production of ROS in macrophages. RAW264.7 cells were treated with PBS, cKp, and hvKp, respectively. The levels of intracellular ROS were quantified using a DCFH-DA fluorescent probe in conjunction with flow cytometry or fluorescence microscope. Interestingly, we found that under the cKp and hvKp strains stimulation, the levels of ROS in macrophages were significantly elevated, while there was only a low level of ROS in the control group (**Figures 2C, D**). Furthermore, hvKp group exhibited remarkably greater ROS production than the cKp group. Taken together, these results suggested that the hvKp strain provoked a more pronounced pro-inflammatory response than the cKp strain and a remarkable increase in ROS levels in macrophages.

### *K. pneumoniae* infection triggers pyroptosis and apoptosis in macrophages

**Figure 3:**
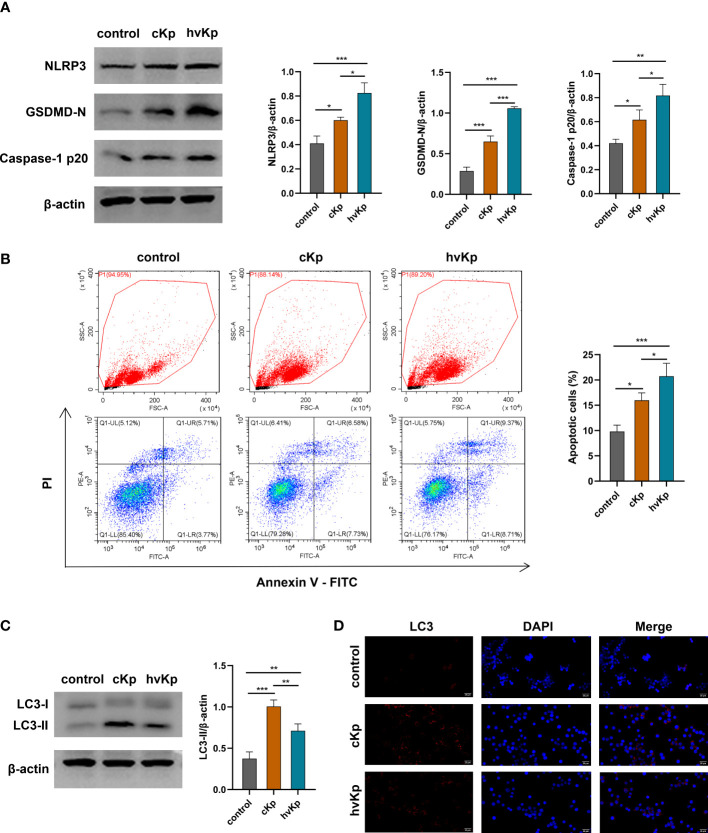
Effects of cKp and hvKp infection on pyroptosis, apoptosis, and autophagy of macrophages. **(A)** The protein expressions of NLRP3, GSDMD-N, and caspase-1 p20 were detected by Western blot and the bands densities were analyzed using Image J. **(B)** The apoptosis of RAW264.7 cells were determined by flow cytometry. FSC/SSC panels were flow gating strategy for apoptosis analysis, Q1-LR (Annexin V+/PI−) was considered as early apoptosis and Q1-UR (Annexin V+/PI+) was considered as late apoptosis in the pseudo color plot. **(C)** The protein levels of LC3 were detected by Western blot and analyzed using Image J. **(D)** The LC3 puncta were detected by immunofluorescence staining (scale bar, 25 μm). All experiments were independently repeated three times. Data are presented as mean ± SD. *p < 0.05, **p < 0.01, ***p < 0.001. cKp, classical *Klebsiella pneumoniae*; hvKp, hypervirulent *Klebsiella pneumoniae*.

We have noted that cKp and hvKp strains prominently promoted the IL-1β production in macrophages. NLRP3 inflammasome is a multi-protein complex comprised of NOD-like receptor NLRP3, the adaptor protein ASC and pro-caspase-1, playing a critical role in the activation of caspase-1 and the secretion of IL−1β. Activated caspase-1 can cleave gasdermin D (GSDMD) into N-terminal pore-forming fragments and C-terminal fragments, ultimately triggering pyroptosis, a form of programmed cell death (Man et al., 2017). To determine whether *K. pneumoniae* infection induces pyroptosis in macrophages, we examined the protein levels of cleaved caspase-1, NLRP3, and GSDMD-N in RAW264.7 cells by Western blot. We observed that *K. pneumoniae* infection upregulated the caspase-1 p20, NLRP3, and GSDMD-N protein levels in macrophages compared with the control group (**Figures 3A**, **S3A-C**). Moreover, significantly higher levels of these proteins were detected in the hvKp group than in the cKp group.

To further examine whether *K. pneumoniae* infection enhances the apoptosis of macrophages, we used flow cytometry to detect the apoptotic cells upon cKp and hvKp infection. As shown in **Figure 3B**, Q1-LR represents the zone of early apoptotic cells (Annexin V+/PI−), and Q1-UR represents the zone of late apoptotic cells (Annexin V+/PI+) in the pseudo color plot; the proportion of apoptotic cells (Q1-LR and Q1-UR) was significantly increased after cKp and hvKp infection, compared with the PBS control group. In addition, the hvKp group exhibited a higher number of apoptotic cells than the cKp group. Taken together, these findings indicated that both cKp and hvKp challenge could induce apoptosis and pyroptosis in macrophages.

### Effects of *K. pneumoniae* infection on autophagy in macrophages

To investigate whether cKp and hvKp strains potentially involved in autophagy of macrophages, we detected the expression of autophagy-related protein LC3-I/II by Western blot. As shown in **Figures 3C**, **S3D** both cKp and hvKp strains could promote LC3-II protein accumulation in macrophages. Meanwhile, the following hvKp treatment reduced the LC3-II protein accumulation compared with the cKp group. Moreover, we further carried out an immunofluorescence analysis to detect the expression of LC3 puncta. Consistent with above results, the LC3 puncta were increased following cKp and hvKp challenge, while LC3 puncta were almost not detected in the control group. Notably, hvKp treatment reduced the increase in LC3 puncta compared with cKp group (**Figure 3D**). Our current results indicated that the hvKp strain may, at least in part, impair autophagy activation in macrophages, in comparison with cKp strain.

### *K. pneumoniae* infection induces acute lung injury in mice

**Figure 4:**
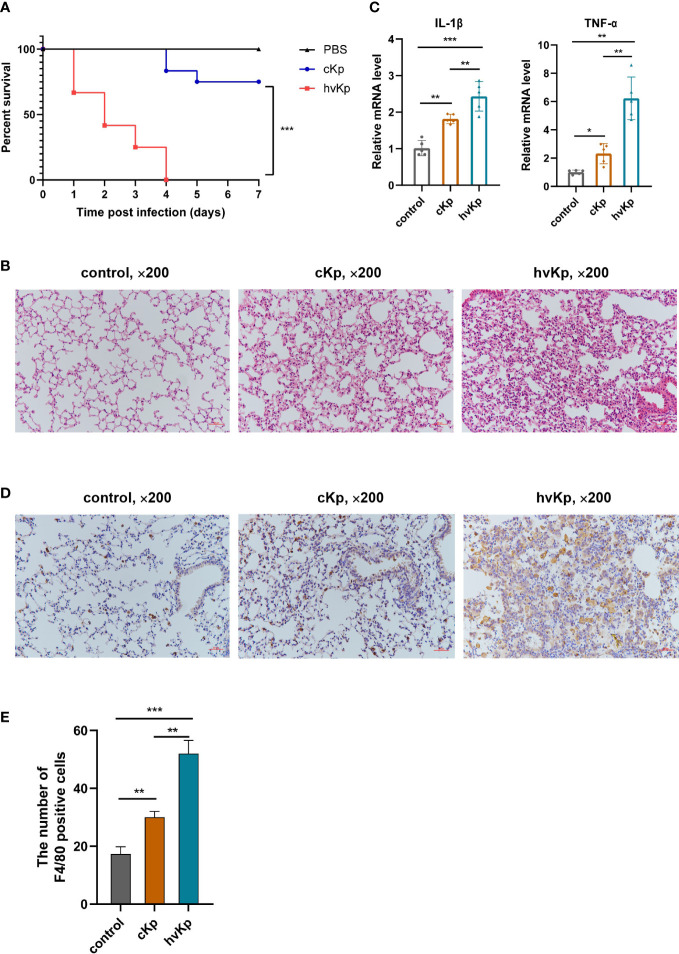
Effects of cKp and hvKp infection on survival and lung injury in mice. **(A)** Survival rates of mice were recorded from day 0 to day 7. **(B)** The pathological changes in lung tissues were determined by H&E staining (×200). **(C)** The mRNA levels of IL-1β and TNF-α in lung tissues were detected by qRT-PCR. **(D)** The macrophages marker F4/80 was detected by immunohistochemical staining (×200). **(E)** Statistical analysis of F4/80 positive cells. Data are presented as mean ± SD. *p < 0.05, **p < 0.01, ***p < 0.001. cKp, classical *Klebsiella pneumoniae*; hvKp, hypervirulent *Klebsiella pneumoniae*.

We established a mouse model of acute pneumonia induced by cKp and hvKp strains, respectively, to further investigate the pathogenicity of the two strains *in vivo*. First, we assessed the survival of mice after infection. Notably, all mice in hvKp group died at 4 days post-infection, while 75% of mice in cKp group remained alive during the entire period of observation (**Figure 4A**). Compared with the cKp strain, the hvKp strain caused a much higher mortality of mice. Pathological changes in the lung tissues were detected using H&E staining. As exhibited in **Figure 4B**, in the control group, mice had clear and intact pulmonary alveolar structures, no inflammatory cell infiltration, no congestion or edema, and no significant thickened alveolar walls. Nevertheless, cKp strain induced moderate lung inflammation, a small amount of inflammatory cell infiltration, and mild thickening of the alveolar walls. By contrast, the hvKp strain triggered a robust pulmonary inflammation. There was extensive inflammatory cell infiltration into alveolar spaces and neighboring tissues, in association with markedly thickened and ruptured alveolar walls, alveolar hemorrhage, and edema. In addition, qRT-PCR results indicated that the mRNA levels of IL-1β and TNF-α in lung tissues were significantly upregulated after cKp and hvKp treatments (**Figure 4C**). Moreover, compared with cKp-infected mice, the levels of these cytokines were much higher in lung tissues following hvKp administration. Immunohistochemistry analysis of F4/80 (a marker of macrophage) revealed that *K. pneumoniae* infection increased the number of macrophages in lung tissues, with a much higher number in hvKp mice (**Figures 4D, E**). Collectively, these data suggested that the hvKp strain can cause more severe lung injury and much higher mouse mortality than cKp.

### *K. pneumoniae* infection regulates multiple cell death pathways *in vivo*

**Figure 5:**
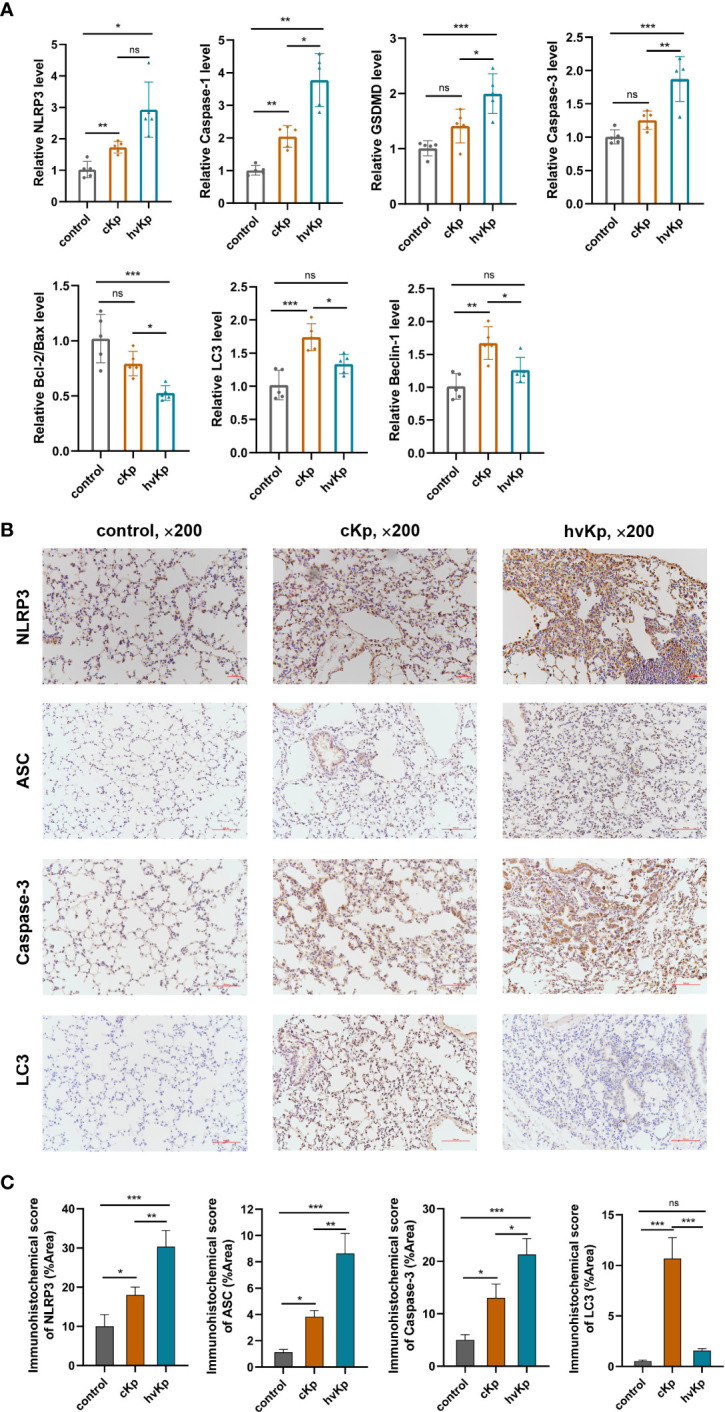
Effects of cKp and hvKp infection on cell death pathways in mice. **(A)** The relative mRNA levels of NLRP3, caspase-1, GSDMD, caspase-3, bcl-2/bax, LC3, and beclin-1 were detected by qRT-PCR. **(B)** Immunohistochemical staining was used to detect the protein expressions of NLRP3, ASC, caspase-3, and LC3 (×200). **(C)** Statistical analyses were performed by ImageJ. Data are presented as mean ± SD. ns, no significance, *p < 0.05, **p < 0.01, ***p < 0.001. cKp, classical *Klebsiella pneumoniae*; hvKp, hypervirulent *Klebsiella pneumoniae*.

To further verify the occurrence of pyroptosis, apoptosis, and autophagy during *K. pneumoniae* infection *in vivo*, we first evaluated the key genes mRNA expression involved in these pathways following *K. pneumoniae* administration in mice. Results showed that *K. pneumoniae* infection upregulated the transcription levels of caspase-3, caspase-1, NLRP3, and GSDMD and downregulated the transcription levels ratio of bcl-2/bax (**Figure 5A**), and the effects were more obvious in hvKp mice than in cKp mice. Additionally, cKp strain dramatically elevated the levels of LC3 and beclin-1 transcription, while hvKp mice manifested relatively lower levels (**Figure 5A**). Furthermore, the protein expressions of caspase-3, NLRP3, ASC, and LC3 in mouse lung tissues were additionally examined by immunohistochemical staining. Consistent with transcription results, hvKp mice exhibited a much higher protein levels of caspase-3, NLRP3, and ASC, but a much lower level of LC3 protein, compared with cKp mice (**Figures 5B, C**). Overall, the results suggested that different cell death pathways including pyroptosis, apoptosis, and autophagy can be activated by *K. pneumoniae* infection to varying degrees *in vivo*.

## Discussion

The innate immune system plays a vital role in the defense against microbial infection. However, many bacteria, including *K. pneumoniae*, have evolved strategies to manipulate cell death and disrupt host defense, resulting in more severe outcomes. Here, we focused on the responses of host cells induced by *K. pneumoniae* infection, mainly including inflammation, cell death, and autophagy.

There was a report that hvKp strains with thick polysaccharide capsules are able to resist neutrophil phagocytosis and complement-mediated killing (Nicolò et al., 2022). Lin et al. (2004) also observed serotype K1/K2 *K. pneumoniae* strains were considerably more resistant to phagocytosis than non-K1/K2 strains. Supporting the above results, our research found that, compared with cKp isolate, engulfment of hvKp isolate by macrophages was significantly lower. Some *Klebsiella* inhibiting phagocytosis would be an important virulence factor (Cano et al., 2015). Next, the viability of infected macrophages was measured by LDH release assay and Calcein-AM/PI double staining. The results showed that hvKp infection triggered a significant decrease in cell viability, and the cytotoxic effect was more pronounced than cKp infection. In the present study, mouse pneumonia models induced by cKp and hvKp strains were established to further investigate the effects of cKp and hvKp infection *in vivo*. From the survival curve results, we discovered that intratracheal administration of hvKp strain dramatically decreased the survival of mice. Additionally, H&E staining revealed that hvKp treatment caused more severe lung tissue damage compared to cKp treatment.

Research conducted previously has shown that infection with *K. pneumoniae* dramatically stimulates the expression of a variety of pro-inflammatory mediators. According to a prior study, the production of TNF-α, COX-2, IL-6, and IL-1β in the murine model was elevated with *K. pneumoniae* stimulation (Lee and Kim, 2011). Consistently, our results showed that hvKp isolate triggers a more serious pro-inflammatory response than cKp isolate *in vitro* and *in vivo*. HvKp treatment resulted in significantly higher mRNA expressions of IL-1β, IL-6, and TNF-α in macrophages and mouse lung tissues than cKp treatment. Regarding cytokine production, ELISA analysis further demonstrated that both cKp and hvKp infection greatly enhanced the release of IL-6, TNF-α, and IL-1β in macrophages. Furthermore, macrophages exposed to the hvKp isolate had a considerably higher IL-1β and TNF-α concentration than those exposed to the cKp isolate. These pro-inflammatory cytokines are released as part of an antimicrobial mechanism that assist in activating the innate and adaptive immune responses, but excessive inflammatory cytokines production can lead to pathological injury and even cause septic shock, multiple organ dysfunction, or tumorigenesis (Arango Duque and Descoteaux, 2014). Nearly all organisms and cells produce ROS, a group of unstable chemicals that mostly include oxygen anions, free radicals, and peroxides (Sies and Jones, 2020). Numerous studies over the past decade have demonstrated that ROS is crucial for regulating various functions of cells responding to a variety of stimuli, including pathogens and endogenous cytokines (Herb and Schramm, 2021; Dong et al., 2022). Consistent with a previous study on *Prototheca zopfii* (Shahid et al., 2017), our findings demonstrated that after *K. pneumoniae* treatment, the generation of intracellular ROS was increased in macrophages. Additionally, the hvKp strain had more obvious effects on ROS accumulation than that of cKp strain. Huge amounts of ROS are generated during the inflammation, which will induce oxidative stress and exacerbate cell injury and cell death through apoptosis, necrosis, and autophagy (Li et al., 2019; Zhang et al., 2021). Thus, we speculated that the more severe infection caused by hvKp is associated with overproduced pro-inflammatory cytokines and ROS in macrophages and lung tissues. The underlying mechanisms associated with cell death need to be further explored.

Pyroptosis is a type of lytic cell death that has been extensively found in infected macrophages, monocytes, and dendritic cells. It usually causes the rupture of cell membranes and the massive releases of damage-associated molecular patterns (DAMPs) and inflammatory cytokines, which in turn strongly aggravates the inflammatory responses, playing a critical role in the anti-microbial immune defense and diseases development (Wu et al., 2015). In the current study, our experiments highlighted that both cKp and hvKp infection induced the occurrence of pyroptosis in macrophages and lung tissues by detecting the protein or mRNA levels of pyroptosis-related genes, including NLRP3, caspase-1, GSDMD, ASC, and IL-1β, while in hvKp infection model, the degree of pyroptosis was more pronounced. This result is consistent with a recent study that *Enterococcus faecalis* CA1 strain with more virulence factors induced a much higher degree of pyroptosis in RAW264.7 cells and a more dramatic macrophage damage compared to *E. faecalis* CA2 strain (Chi et al., 2021). Our results revealed that *K. pneumoniae* infection can lead to cellular pyroptosis depending on the virulence of strains. Excessive pyroptosis caused by hvKp infection promotes cellular and tissue damage in the present study; as reported by Dong et al. (2021), PSPA treatment can alleviate pulmonary inflammation and injury in *K. pneumoniae*-infected mice by inhibiting pyroptosis. Excessive pyroptosis is not only detrimental to host defense but also triggers strong inflammatory responses to exacerbate tissue damage (Wang et al., 2020a). A previous study reported that *P. gingivalis*-derived OMVs can activate caspase-1 and induce the release of inflammatory cytokines and pyroptotic cell death in macrophages (Fleetwood et al., 2017). OMVs secreted from Gram-negative bacteria play a significant role in bacterial pathogenicity by delivering virulence factors and other pathogen-associated molecular patterns (PAMPs) into the host cells. You et al. (2020) found that OmpA (present in OMVs) of *K. pneumoniae* ATCC 13883 exerted oxidative stress and promoted pyroptosis and apoptosis in HEp-2 cells, which resulted in the death of host cells. It has been reported that the production of OMVs of hvKp was much higher compared to cKp under iron-deficient environment (Lan et al., 2022). Thus, we speculated that OMV-derived from hvKp may contribute to the cell death induced by hvKp in the present study. However, further studies are essential to identify the roles of OMVs in the pathogenicity of hvKp. A recent study showed that *K pneumoniae* could induce colitis and accelerate DSS-mediated colitis through the release of mature IL-18 mediated by caspase-11 in the gut epithelial cells (Zhang et al., 2023). As a non-canonical inflammasome, caspase-11 can cleave GSDMD to release its N-terminal domain and in turn induce pyroptosis by direct recognition of intracellular LPS (Kayagaki et al., 2011). Whether caspase-11 activation varies in defense against cKp and hvKp infection in the present study requires further investigation. Previous research suggested that ROS can positively regulate the activation of NLRP3 inflammasome, and the NLRP3 inflammasome activity was inhibited by the ROS inhibitor in LPS-activated macrophages (Tschopp and Schroder, 2010; Zhou et al., 2011). Zhou et al. (2010) showed that direct simulation of THP-1 cells with hydrogen peroxide can activate NLRP3 inflammasome, which in turn triggers the activation of caspase-1 and the release of IL-1β. In the current study, the ROS production may be responsible for the NLRP3 inflammasome activation, but the relationship between the two needs to be investigated in the future.

Different from pyroptosis, apoptosis is a form of cell death with a less inflammatory response. In our study, flow cytometry analysis revealed a considerable rise in apoptotic cells with *K. pneumoniae* treatment. It is noteworthy that following hvKp infection, there were more apoptotic cells than cKp infection. Moreover, the *in vivo* results also indicated that the hvKp strain can significantly increase the tissue apoptosis compared to cKp strain. This result is consistent with pathogenic *L. interrogans* that could induce apoptosis and necroptosis in myeloid neutrophils and monocytes at 24 and 72 h post-infection, ultimately leading to a chemo-cytokine storm-mediated robust inflammatory response (Shetty et al., 2021; Kundu et al., 2022). It was found that M1 but not M2 macrophages are more susceptible to inflammation-related necrotic cell death, which may be associated with the overexpression of key necroptosis signaling molecules such as RIPK3, MLKL, and ZBP1. Strong necroptosis occurring in M1 macrophages may act as an inflammatory amplifier and play a key role in the development of acute infection and tissue damage (Hao et al., 2021). It is generally acknowledged that apoptosis usually results in the resolution of inflammation, whereas cell necrosis can lead to persistent inflammation and prolong the disease. Thus, immune cells continue to contribute to the inflammatory process even after they die, enabling the host to strengthen the immune response (Kundu et al., 2022). Our results showed that *K. pneumoniae* infection caused apoptosis and inflammatory necrosis in macrophages, but inflammation was unresolved and infection became worse. Excessive cell death is usually detrimental to the host, with the massive death of immune cells leading to bacteria to disseminate and infect other cells. As reported by Zhang et al. (2021), *Salmonella typhimurium* induced macrophages death, resulting in a decrease in the bactericidal ability and subsequently a robust inflammatory action. Therefore, these findings suggested that excessive removal of macrophages from the innate immune system and increased inflammation induced by pyroptosis may contribute to the high pathogenicity of hvKp. Furthermore, macrophages can produce numerous mediators to recruit neutrophils to infection sites, and the interaction of neutrophils and macrophages can also be crucial for pathogen clearance. As reported by a recent study, Runx3 general KO dramatically increased the numbers of macrophages and neutrophils and the production of pro-inflammatory cytokines for combating IAV infection, hence compensating for the loss of pulmonary CD8+ T cells following IAV infection (Hao et al., 2022). Moreover, neutrophil-mediated responses are demonstrated to be important for initial control of the infection in the mouse models induced by *K. pneumoniae* (Batra et al., 2012). Previous studies showed that hypervirulent *K. pneumoniae* infection delayed neutrophils apoptosis, which resulted in impaired efferocytic clearance of infected neutrophils by macrophages. Efferocytosis is a protective process in which macrophages recognize specific “eat-me” signals on apoptotic cells and engulf dead cells, thereby controlling bacterial transmission and resolving inflammation and infection. Impaired efferocytosis can initiate secondary necrosis and lead to bacterium spread, autoimmunity, and persistent inflammation (Lee et al., 2017a; Jondle et al., 2018). Studies on the effects of hvKp infection on macrophage apoptosis are extremely limited. Our findings demonstrated that hvKp infection can promote the apoptosis of macrophages, which may further impede the efferocytosis with the reduction in macrophage numbers. Additionally, other studies have shown that *K. pneumoniae* can promote apoptosis of platelets, bMEC cells, and bronchial epithelial cells (Wang et al., 2018; Cheng et al., 2020; Jiang et al., 2022). The types and degrees of cell death in the interaction of host cells and pathogens vary depending on the pathogen type, infection dose, and infected target. Due to the diverse immunological reactions, the same pathogen can cause different modes of cell death in different cell types.

Without doubts, autophagy generally supports cell survival in most cases (Kroemer et al., 2010). Two highly conserved macrophage processes, phagocytosis and autophagy, are essential to the innate immune defense against bacterial infection. Accumulating evidence suggests that autophagy can regulate a variety of macrophage functions, such as phagocytosis. For example, Jati et al. (2018) reported that Wnt5A-Rac1-Disheveled signaling-mediated actin assembly directs the clearance of *P. aeruginosa* and *S. pneumoniae* strains in macrophages through phagocytosis and subsequent xenophagy (a selective autophagy targeting invasive pathogen). Coincidentally, Xu et al. (2020) found that *T. pallidum* stimulates macrophage autophagy to enhance phagocytosis and clearance. In addition, several studies have suggested that autophagy can reduce oxidative damage by decreasing ROS production (Li et al., 2015a). Maurer et al. (2015) also reported increased cell death induced by *S. aureus* a-toxin occurs in mouse endothelial cells upon autophagy inhibition. Thus, we further examined the autophagy levels involved in *K. pneumoniae* infection. LC3 and beclin-1 are specific biochemical markers of autophagy (Zhang et al., 2015). In our study, we used fluorescence microscopy, qRT-PCR, and Western blot to show that autophagy occurs both *in vitro* and *in vivo*. Surprisingly, the expression of LC3 and beclin-1 was high in the cKp group but was relatively low in the hvKp group, showing that cKp greatly triggers autophagy while hvKp fails to induce autophagy. This result is in line with hypervirulent *K. pneumoniae* (ATCC 43816) infection impaired autophagy flux and resulted in an insufficient autophagy response, ultimately leading to severe lung injury in mice (Nikouee et al., 2021). Therefore, we infer that failing to induce autophagy partly may be the reason why hvKp infection causes more serious damage of cells and organs in the present study. This hypothesis can be further supported by a recent study that therapy with TB-peptide can significantly alleviate the adverse outcomes of *K. pneumoniae*-induced sepsis by enhancing autophagy (Nikouee et al., 2021). Furthermore, Atg7 deficiency in macrophages impaired host defense against *K. pneumoniae*, resulting in attenuated bacterial clearance and worsened lung injury of mice (Ye et al., 2014). Of note, just as the above results show that hvKp can resist phagocytosis, further studies are required to investigate whether failing to induce autophagy by hvKp leads to the resistance of macrophage-mediated phagocytosis, although CPS plays a major role in the anti-phagocytosis of hvKp.

One limitation in this research is that only two *K. pneumoniae* strains were compared, and these two strains represented cKp strain and hvKp strain, respectively. However, we know that *K. pneumoniae* isolates contain a variety of different serotypes and virulence profiles, and it is unclear how representative these two strains are. A study in the future that includes more *K. pneumoniae* isolates is needed. Therefore, we emphasize that our study was a preliminary exploration of the mechanisms of the interactions between the host cells and *K. pneumoniae*.

## Conclusion

In summary, we demonstrated that the hvKp strain was more resistant to phagocytosis by macrophages compared with cKp strain, but significantly reduced cell viability *via* promoting ROS production and pro-inflammatory response and, at least partly, failing to induce autophagy, eventually leading to cell death through pyroptosis and apoptosis. These results mentioned above were further confirmed *in vivo*. Importantly, our research shed new light on the pathogenesis of *K. pneumoniae* and may contribute to develop new approaches in the treatment of *K. pneumoniae* infection in the future.

## Data availability statement

The original contributions presented in the study are included in the article/**Supplementary Material**. Further inquiries can be directed to the corresponding author.

## Ethics statement

The animal study was reviewed and approved by the Laboratory Animal Welfare Ethics Committee of Qingdao University.

## Author contributions

XW, CB and ZY contributed to conception and design of the study. XW carried out the experiments and wrote the first draft of the manuscript. XW and XX performed the statistical analysis. MZ, HF, LL and MW carried out the experiments. All authors contributed to manuscript revision, read, and approved the submitted version.
