# Supplementary material for: Pyroptosis, apoptosis, and autophagy are involved in infection induced by two clinical Klebsiella pneumoniae isolates with different virulence
Source: Front Cell Infect Microbiol. 2023 May 8;13:1165609. doi: 10.3389/fcimb.2023.1165609 (PMC10200925; doi:10.3389/fcimb.2023.1165609)
Supplement: Supplementary file 1 [file DataSheet_1.docx]

Supplementary Material

**Supplementary Table**

**Table S1.** Characteristics of *K. pneumoniae* strains used in this study

|  | String test | capsular type | |  | genetic marker | | |
| --- | --- | --- | --- | --- | --- | --- | --- |
|  |  | K1 | K2 |  | rmpA | iucA | peg-344 |
| cKp | - | - | - |  | - | - | - |
| hvKp | + | - | + |  | + | + | + |
| cKp: classical *K. pneumoniae*; hvKp: hypervirulent *K. pneumoniae* | | | | | | | |

**Supplementary Figures**

A B


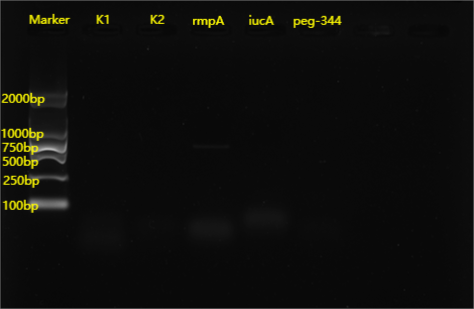

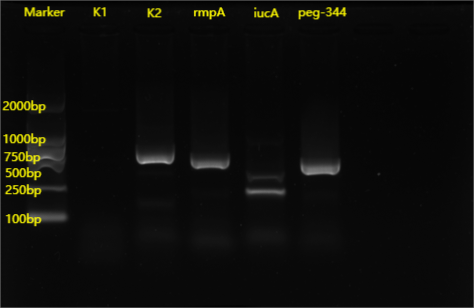


**Figure S1.** The results of genetic marker of K1, K2, rmpA, iucA, and peg-344 by PCR. (A) cKp strain. (B) hvKp strain.


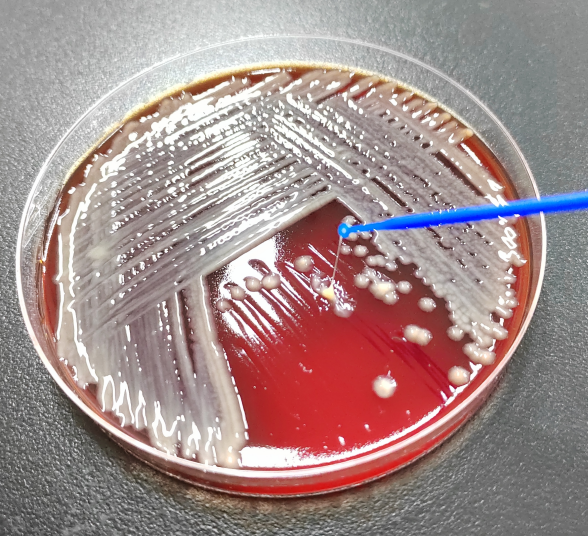


**Figure S2.** The positive result of string test.

A


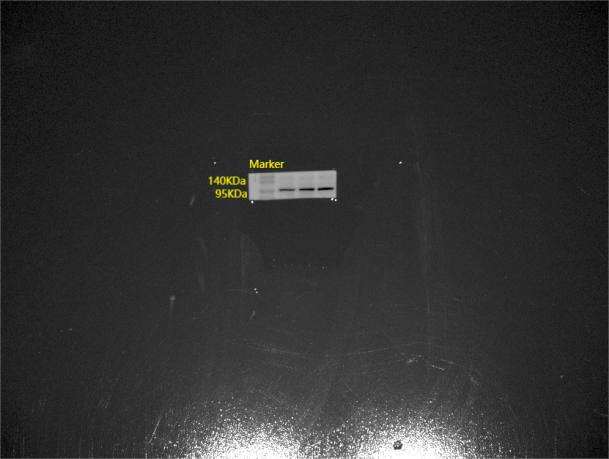

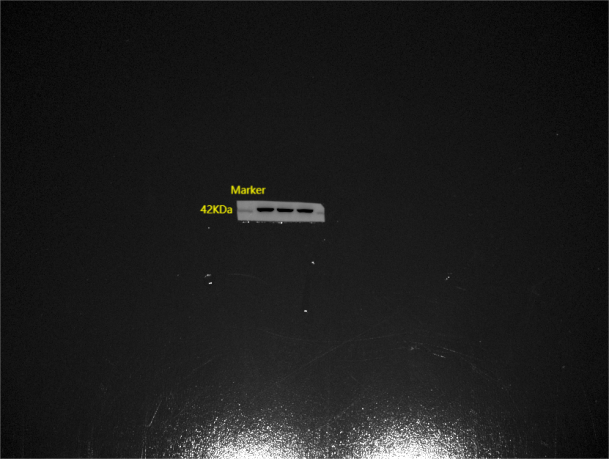


B


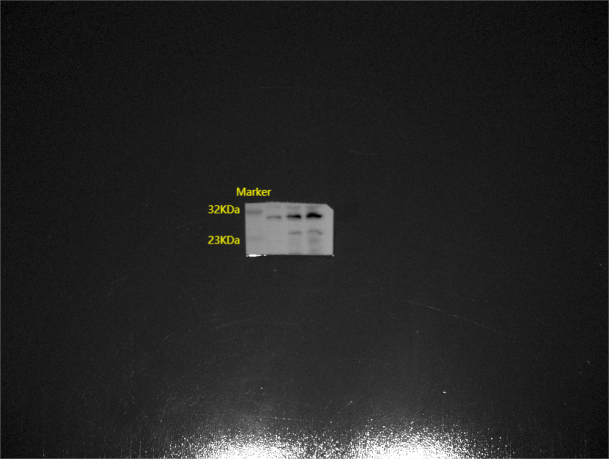

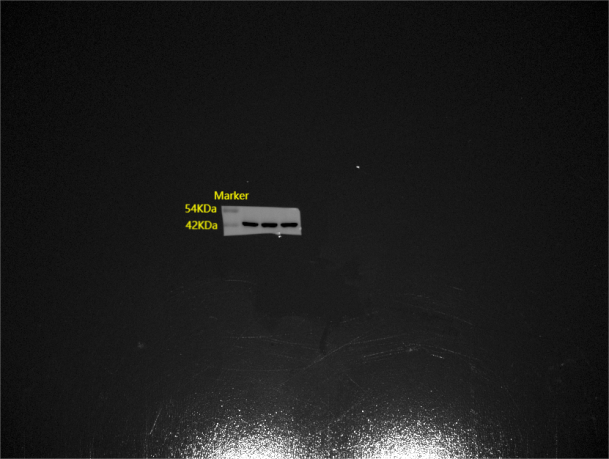


C


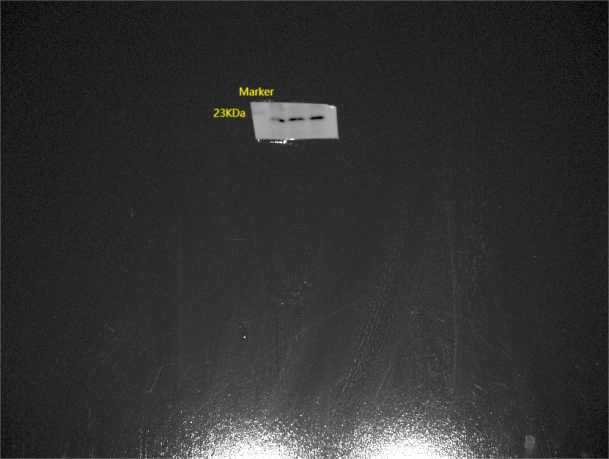

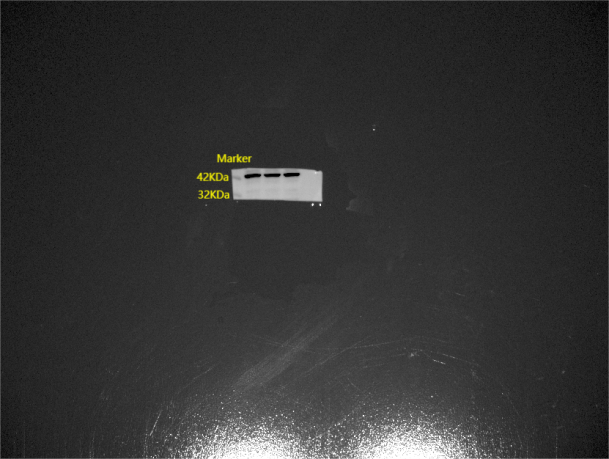


D


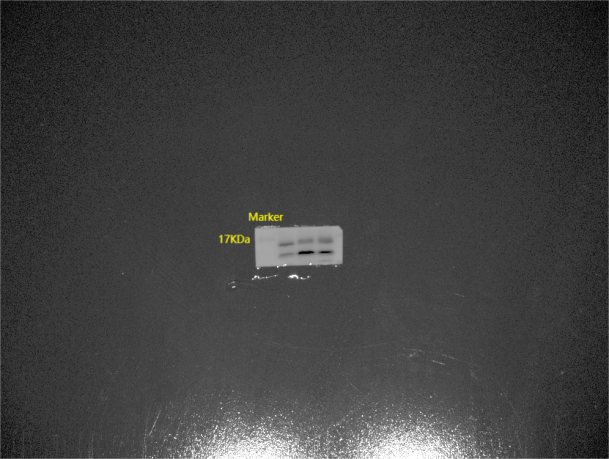

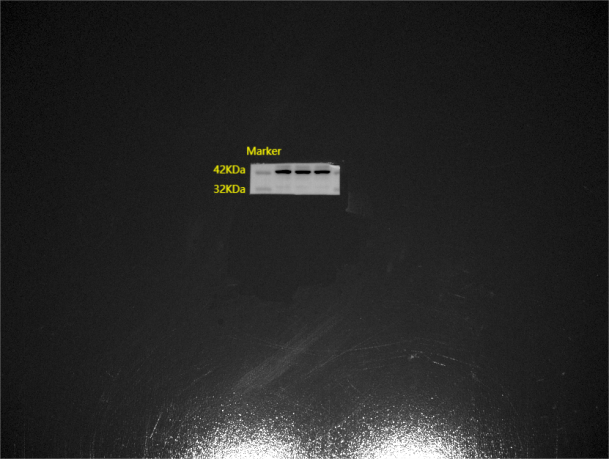


**Figure S3.** The protein expression of NLRP3, GSDMD-N, caspase-1 p20, and LC3 and corresponding β-actin were detected by Western blot. (A) NLRP3 and corresponding β-actin. (B) GSDMD-N and corresponding β-actin. (C) caspase-1 p20 and corresponding β-actin. (D) LC3 and corresponding β-actin.
